# Views about primary care health checks for autistic adults: UK survey findings

**DOI:** 10.3399/BJGPO.2022.0067

**Published:** 2022-09-07

**Authors:** David Mason, Helen Taylor, Barry Ingham, Tracy Finch, Colin Wilson, Clare Scarlett, Anna Urbanowicz, Christina Nicolaidis, Nicholas Lennox, Sebastian Moss, Carole Buckley, Sally-Ann Cooper, Malcom Osborne, Deborah Garland, Dora Raymaker, Jeremy R Parr

**Affiliations:** 1 Population Health Sciences Institute, Newcastle University, Newcastle upon Tyne, UK; 2 Cumbria Northumberland Tyne and Wear NHS Foundation Trust, Morpeth, UK; 3 Department of Nursing, Midwifery and Health, Northumbria University, Newcastle upon Tyne, UK; 4 NHS Newcastle Gateshead Clinical Commissioning Group, Newcastle upon Tyne, UK; 5 NHS North Tyneside Clinical Commissioning Group, Newcastle upon Tyne, UK; 6 Queensland Centre for Intellectual and Developmental Disability, MRI-UQ, The University of Queensland, Brisbane, Queensland, Australia; 7 Social and Global Studies Centre, School of Global, Urban and Social Studies, RMIT University, Melbourne, Victoria, Australia; 8 School of Social Work, Portland State University, Portland, Oregon, USA; 9 Department of Medicine, Oregon Health and Science University, Portland, Oregon, USA; 10 NHS Northumberland Clinical Commissioning Group, Morpeth, UK; 11 Royal College of General Practitioners, London, UK; 12 Institute of Health and Wellbeing, University of Glasgow, Glasgow, UK; 13 The Kayaks support group, South Shields, UK; 14 National Autistic Society, London, UK; 15 Regional Research Institute for Human Services, Portland State University, Portland, Oregon, USA

**Keywords:** inequalities, health promotion and prevention, clinical (general), autistic disorder, primary health care, general practice

## Abstract

**Background:**

Compared with the general population, autistic adults experience higher rates of physical and mental health conditions, premature morbidity and mortality, and barriers to health care. A health check for autistic people may improve their health outcomes.

**Aim:**

To establish the views of autistic people towards a primary care health check for autistic people.

**Design & setting:**

Cross-sectional questionnaire study in England and Wales.

**Method:**

A questionnaire was sent to autistic adults with physical health conditions in England and Wales. A total of 458 people (441 autistic adults and 17 proxy responders) completed the questionnaire.

**Results:**

Most responders (73.4%, *n* = 336) thought a health check is needed for all autistic people. Around half of the participants thought a health check should be offered from childhood and the health check appointment should last between 15 and 30 minutes. Autistic people were positive about providing primary care staff with contextual information regarding their health and the reasonable adjustments they would like before their health check appointment. Training about autism and the health check was considered important, alongside adequate time for discussions in the health check appointment (all by over 70% of responders). The clinician’s autism knowledge, seeing a familiar clinician, environmental adaptations, appropriate information, and accessible appointments were considered particularly important in making a health check accessible.

**Conclusion:**

Autistic people and relatives were supportive of a primary care health check for autistic people. Information gathered was used to support the design of a primary care health check for autistic adults.

## How this fits in

Compared with the general population, autistic adults experience higher rates of health conditions, premature morbidity and mortality, and barriers to effective health care. Strong support was found for a primary care health check for autistic adults, and important information was given about health check design and delivery. Most responders thought the health check should be available to all autistic adults. Autistic people thought consideration of personalised reasonable adjustments would improve acceptability and access to the health check.

## Introduction

Autism research has started to systematically investigate co-occurring mental and physical health conditions, and healthcare access for autistic people.^1,2^ Large scale studies have found an increased prevalence of health conditions in autistic people versus population samples, independent of whether intellectual disability is present.^3–7^ For example, autistic adults seem more likely to experience premature mortality from neoplasms, endocrine conditions, and conditions of the nervous, circulatory, and respiratory systems.^8,9^

Studies highlight barriers faced by autistic adults accessing health care including the following: waiting areas that cause sensory overload;^10,11^ problems with patient–provider communication;^12^ a lack of training about autism among healthcare providers;^13^ and cognitive difficulties.^14,15^ For example, difficulties with executive functioning impacting on follow-up of care, missing appointments owing to memory difficulties, and translating medical care into concrete actions.^15–17^ These factors may increase distress throughout the healthcare experience (for example, when travelling to healthcare appointments, waiting for and during the appointment) that only reduces after returning home.^11^ Barriers likely contribute to difficulties identifying and treating autistic adults’ health conditions. In the UK, adjustments to healthcare service provision may be available; however, a recent study showed a gap between adjustments autistic people want from services and what was received.^18^

Annual health checks specifically designed for people with intellectual disability have been developed and evaluated in randomised controlled trials.^19,20^ In England, a health check for people with intellectual disabilities has been integrated into primary care. A review of health checks for people with intellectual disabilities found they consistently detected unmet health needs and led to targeted actions to address needs.^21^ Autistic people with co-occurring intellectual disabilities may receive a health check for people with intellectual disabilities; however, this health check is not available to (and may not be appropriate for) the significant proportion of autistic people who do not have co-occurring intellectual disabilities. Recent healthcare policy in England has advocated for the development and evaluation of health checks specifically designed to meet the needs of all autistic people (*NHS Long Term Plan* and *National Strategy for Autistic Children, Young People and Adults*).^22,23^ A health check for all autistic adults that incorporates adjustments to health care may help to overcome barriers to access, reduce health inequalities, improve health, and reduce early mortality for autistic people with and without intellectual disability.

This health checks research programme was conceived at a workshop to establish priority research areas to improve the physical health and wellbeing of autistic people.^24^ The research aimed to investigate the views of autistic people about a specifically designed health check, the important features of design and delivery, and what would make accessing a health check easier (or more challenging). The study methods and materials were co-designed with autistic people and relatives as part of a wider programme of co-produced research (https://research.ncl.ac.uk/autismhealthchecks).

## Method

### Participants

Recruitment was through the Adult Autism Spectrum Cohort-UK (ASC-UK),^25^ an ongoing, longitudinal study of the lived experiences of UK autistic adults; any UK-based autistic person aged ≥16 years is able to participate in ASC-UK. Cohort recruitment was through any source, including health providers, voluntary sector, and community organisations. All ASC-UK participants aged ≥18 years who provided baseline data identifying they had one or more physical health condition (for example, diabetes, hypertension, asthma, arthritis), and lived in England or Wales were eligible to be contacted. All autistic adults with capacity to do so had given consent to be re-contacted; autistic adults who could not give informed consent for themselves were represented by a relative or carer authorised to act on their behalf (termed a ‘proxy responder’).^25,26^

### Materials

The research team (which includes autistic people) designed the survey, and autistic people who were not part of the study team were consulted about content and piloted the survey. Responders' demographic information was available from ASC-UK (data access supported by ASC-UK chief investigator Dr Jeremy Parr). The survey included the following: items about a health check for autistic adults, including provision (*Do you think regular health checks for autistic people should be provided?*); and delivery (*At what age do you think health checks should be offered? How often should they be carried out? How long would you expect a health check for autistic people to last? How would you like to be told what to expect from a health check specifically for autistic adults? Would you be happy to provide information about your health needs before the health check? Would you be happy to provide information about any reasonable adjustments you’d like before the health check?*). Questions also focused on health check delivery and implementation (*What would make health checks easier for autistic adults or harder for autistic adults?*).

Most questions offered fixed-choice responses; there were opportunities for free-text responses (for example, to elaborate on reasons for response selection or add information). Participants were asked to provide up to three free-text responses regarding what would make health check access easier and three free-text responses regarding what would make a health check harder).

### Procedure

Eligible participants were sent an information sheet and survey by post or email. The electronic version of the survey was hosted on Qualtrics.^27^ Participants could contact the research team for assistance completing the questionnaire (one participant made use of this). A reminder was sent to eligible participants after 1 month.

### Analysis

Data from autistic people and proxy responders were grouped together for analysis.

To investigate representativeness, responders and non-responders were compared (using χ^2^ tests) on age (18–25 years, 26–40 years, 41–60 years, ≥61 years), sex (male and female), and preferred contact method (post or email).

Summary statistics were calculated for closed survey questions. Several questions allowed for multiple options to be selected, meaning that the number of responses could be greater than the number of participants. To explore systematic differences in responses, comparisons were made by the following: sex (male and female, 14 participants did not report a sex of male or female and were excluded from sex comparisons only); by age; and by intellectual disability. Post-hoc comparisons were not carried out because there were insufficient participants within groups.

Content analysis was completed for free-text responses; an inductive approach was used.^28^ HT read responses and created categories to organise data. HT and DM separately coded data, assigning responses to categories. Any differences in categorisation were discussed, and agreement reached. Where responses did not fit into a category, further categories were created. HT and DM further reviewed the categories and condensed these into 17 final categories. Each response from each participant was coded separately. The number of responses within each category were expressed as a proportion of the total responses (excluding non-codable responses). Non-codable responses included: expressions of uncertainty; ambiguous responses not attributable to a category, such as 'pre-conceptions' or 'lack of knowledge'; answers unrelated to the topic; or answers stating no opinion.

## Results

A total of 1001 people (944 autistic adults and 57 proxy responders) were contacted and 458 people consented to take part (46%*, n* = 441 autistic adults, and *n* = 17 proxy responders). Participant characteristics are described in Table 1.

**Table 1.** Participant characteristics

Characteristic	Frequency, *n* (%)
Total participants	458
Age, years	
18–25	47 (10.3)
26–40	148 (32.3)
41–60	202 (44.1)
≥61	61 (13.3)
Sex	
Male	183 (40.0)
Female	244 (53.3)
Did not report as male or female^a^	14 (3.0)
No response	17 (3.7%)
Qualifications achieved^b^	
Postgraduate degree	74 (16.2)
Bachelor’s degree	163 (35.6)
Diploma of higher education	59 (12.9)
Certificate of higher education	49 (10.7)
A-level or equivalent	245 (53.5)
General Certificate of Secondary Education (GCSE)	341 (74.5)
Basic skills	87 (19.0)
No formal qualifications	39 (8.5)
Other	76 (16.6)
Employment status^b^	
Employed without support	142 (31.0)
Employed with support	6 (1.3)
Self-employed	36 (7.9)
Volunteer	58 (12.7)
Unemployed	194 (42.4)
Retired	25 (5.5)
Other	78 (17.0)
Autism diagnosis	
Formal diagnosis	392 (85.6)
Suspected or unsure or awaiting assessment	66 (14.4)
Mean age at diagnosis (SD; range)	37.7 (15.6; 2.0–68.0)
SRS score by severity category^c^	
Normal	9 (2.3)
Mild	38 (9.6)
Moderate	121 (30.6)
Severe	227 (57.5)
No response	63 (13.8%)
Mean (SD)	116 (26)
Intellectual disability^d^	
Formal diagnosis	28 (6.1)
Suspected	33 (7.2)
Type of secondary school attended^b^	
Mainstream school	424 (92.6)
Specialist schooling (at any time)	54 (11.8)
Statement of special educational needs	
Yes	60 (13.1)
No	362 (79.0)
Don’t know	32 (7.0)
No response	4 (0.9)
Additional help with learning received at mainstream school^b^	
No help	349 (76.2)
Classroom support	35 (7.6)
One-to-one instruction	22 (4.8)
Extra time for exams	53 (11.6)
Special equipment	13 (2.8)
Quiet room	28 (6.1)
Support person	36 (7.9)
Support received currently^b^	
Home	135 (29.5)
Employment	45 (9.8)
Health	117 (25.5)
Finance	120 (26.2)
Social	67 (14.6)
Lifelong learning	56 (12.2)
Community	86 (18.8)
Organisation	70 (15.3)
Do not receive support	205 (44.8)
Support needed currently^b^	
Home	120 (26.2)
Employment	190 (41.5)
Health	161 (35.2)
Finance	202 (44.1)
Social	247 (53.9)
Lifelong learning	222 (48.5)
Community	136 (29.7)
Organisation	140 (30.6)
Do not want to receive support	61 (13.3)
Completion of survey	
On own	371 (81.0)
With help from somebody else	77 (16.8)
No response	10 (2.2)

^a^Not included in analyses of sex comparison; data included in all other analyses. ^b^Responders could tick more than one option, so percentages add up to more than 100%. ^c^The Social Responsiveness Scale (SRS) scores are reported under the categories given by the SRS authors; however, the authors are aware that some autistic people may dislike this terminology and view it as pathologising. ^d^Responders were asked to tick the relevant box if they had been diagnosed with or suspected they had an intellectual disability.

### Responders and non-responders

Responders and non-responders were rather similar; nevertheless, owing to group size, there were some statistically significant differences between the groups (see Table 2).

**Table 2.** Comparison of survey responders and non-responders on age group, sex, and preferred contact method

Variable	Responder,*n* (%)	Non-responder,*n* (%)	χ^2^,(*P* value)	Effect size,Cramer’s V
Age, years				
18–25	47 (10.3)	78 (14.4)	16.35 (0.001)	0.128
26–40	148 (32.3)	211 (38.9)		
41–60	202 (44.1)	214 (39.4)		
≥61	61 (13.3)^a^	40 (7.4)^a^		
Sex				
Male	183 (40.0)	247 (49.1)	6.15 (0.046)	0.081
Female	244 (53.3)	246 (48.9)		
Did not report as male or female^b^	14 (3.0)	10 (2.0)		
Preferred contact method				
Post	111 (24.3)	93 (17.3)	7.35 (0.007)	0.086
Email	346 (75.7)	444 (82.7)		

^a^Indicates significant differences, based on χ^2^ residuals.^b^Not included in analyses of sex comparison; data included in all other analyses

### Quantitative responses

Responses relating to the importance and content of the health check are summarised in Table 3.

**Table 3.** Proportion of responses to quantitative survey questions about important aspects of the health check from autistic adults, with sex and age effects

	Yes*n* (%)	No*n* (%)	Don’t know*n* (%)	No response*n* (%)	Sex effectsCramer’s V(*P* value)	Age effectsCramer’s V(*P* value)	Intellectual disability effects Crammer’s V(*P* value)
Do you think that regular health checks should be provided for autistic adults?^a^	336 (73.4)	23 (5.0)	55 (12.0)	44 (9.6)	0.06 (.557)	0.21 (0.009)*	0.13 (0.033)*
18–25 years	33 (94.3)	0 (0.0)	2 (5.7)				
26–40 years	104 (83.2)	9 (7.2)	12 (9.6)				
41–60 years	143 (78.6)	6 (3.3)	33 (18.1)				
≥61 years	43 (78.2)	7 (12.7)	5 (9.1)				
Would you be happy to provide information about your health needs before the health check?	398 (86.9)	7 (1.5)	0 (0)	53 (11.6)	0.02 (0.689)	0.04 (0.877)	0.01 (0.850)
Would you be happy to provide information about any reasonable adjustments you’d like before the health check?	395 (86.2)	9 (2.0)	0 (0)	54 (11.8)	0.01 (0.796)	0.12 (0.147)	0.01 (0.916)
**Importance ratings for features of a health check for autistic adults:**	**Not important** ***n*** **(%)**	**Somewhat important** ***n*** **(%)**	**Very important** ***n*** **(%)**	**No response** ***n*** **(%)**			
All staff receiving training about autism	2 (0.4)	39 (8.5)	365 (79.7)	52 (11.4)	0.15 (0.011)*	0.12 (0.506)	0.03 (0.819)
Males	2 (1.3)	23 (14.5)	134 (84.3)				
Females	0 (0.0)	15 (6.8)	206 (93.2)				
Training about health checks for autistic adults	9 (2.0)	73 (15.9)	324 (70.7)	52 (11.4)	0.09 (0.245)	0.18 (0.050)	0.06 (0.520)
An online health check for autistic adults	72 (15.7)	144 (31.4)	182 (39.7)	60 (13.1)	0.08 (0.275)	0.15 (0.170)	0.02 (0.908)
A paper version of the health check	59 (12.9)	149 (32.5)	191 (41.7)	59 (12.9)	0.06 (0.489)	0.15 (0.200)	0.08 (0.284)
Having adequate time for discussions	3 (0.7)	49 (10.7)	353 (77.1)	53 (11.6)	0.11 (0.100)	0.18 (0.056)	0.07 (0.341)

Statistically significant differences are marked in bold and with asterisk.

It was found that 73.4% of participants (*n* = 336) thought that a regular health check should be provided, with only 5.0% (*n* = 23) not wanting a regular health check, and 12.0% (*n* = 55) being unsure. There were no differences in these proportions for sex (all χ^2^ *P* values ≥0.05), but there was a significant effect of age and intellectual disability. As part of a health check, the majority of participants (86.9%, *n* = 398) were willing to provide information about their health needs. Similarly, 86.2% of responders (*n* = 395) said they would provide information on the reasonable adjustments they would like. There were no age, sex, or intellectual disability effects associated with responses to these items (see Tables 3 4).

**Table 4.** Proportion of responses to quantitative survey questions regarding the health check

	Yes,*n* (%)	Sex effects,Cramer’s V (*P* value)	Age effects,Cramer’s V (*P* value)	Intellectual disability effects Crammer’s V (*P* value)
Who should a health check be for?		0.04 (0.880)	0.23 (0.030)*	0.12 (0.173)
All autistic adults	311 (67.9)			
Only autistic adults without ID	9 (2.0)			
Only autistic adults with ID	14 (3.1)			
Don’t know or unsure	39 (8.5)			
Missing	85 (18.6)			
At what age should health checks be provided?		0.11 (0.683)	0.27 (0.050)	0.15 (0.147)
From childhood	259 (56.6)			
From teenage (≥14 years)	49 (10.7)			
From early adulthood (≥18 years)	55 (12.0)			
From middle age (≥40 years)	12 (2.6)			
From older age (≥50 years)	12 (2.6)			
All	11 (2.4)			
Missing	60 (13.1)			
How often should an autism-specific health check be carried out?		0.12 (0.214)	0.21 (0.134)	0.10 (0.593)
Annually	281 (61.4)			
Every 2–3 years	59 (12.9)			
Every 4–5 years	16 (3.5)			
Other	53 (11.6)			
Multiple selections	5 (1.1)			
How long would you expect an autism-specific health check to last?		0.03 (0.971)	0.15 (0.518)	0.06 (0.705)
<15 minutes	29 (6.3)			
15–30 minutes	232 (50.7)			
31–45 minutes	92 (20.1)			
>45 minutes	45 (9.8)			
Missing	60 (13.1)			
How would you like to be told about an autism-specific health check?		0.501	0.20 (0.422)	0.14 (0.135)
By letter	250 (54.6)			
By text	16 (3.5)			
By phone	5 (1.1)			
By email	103 (22.5)			
I would not want to be told in advance	4 (0.9)			
Other	29 (6.3)			
Missing	51 (11.1)			

ID = Intellectual disabilities. Statistically significant results are marked in bold with asterisk.

Responses showed a clear preference for primary care staff to be trained in autism, and delivering a health check for autistic people (rated ‘very important’ by *n* = 365 responders (79.7%) *and n* = 324 responders (70.7%) respectively). Autistic people thought that a health check should be available online and on paper, with only a minority thinking this was not important (*n* = 72, 15.7% for online; *n* = 59, 12.9% for paper). Finally, over-three quarters (77.1%, *n* = 353) thought having adequate time for discussions was ‘very important’ (Table 3). Although there were no age or intellectual disability effects associated with these responses, there was a sex effect when participants were asked about the need for all staff involved in the health check to receive training about autism.

Most people (67.9%, *n* = 311) wanted a health check to be for all autistic people, irrespective of whether they had intellectual disabilities. Only nine responders (2.0%) reported that it should only be for people without intellectual disability. There were no differences in these proportions for sex (all χ^2^ *P*-values ≥0.05), or intellectual disability, but there was a significant effect of age.

67.3% of responders (*n* = 308) thought a health check should be available from childhood or the teenage years; 5% (*n* = 24) thought health checks should start from age ≥40 years.

There was a strong preference for an annual health check (61.4%, *n* = 281). Half of the responders (50.7%, *n* = 232) thought a health check should last for 15–30 minutes (options ranged from <15 minutes to >45 minutes). Just over half of responders wanted to be contacted about a health check by letter (54.6%, *n* = 250). There were no age, sex, or intellectual disability effects associated with these responses.

### Open-text responses and categories

#### Inter-rater reliability

Cohen’s Kappa was used to estimate inter-rater reliability. For both open-text questions, percentage agreement and Kappa statistic were high. For the facilitators question data, Cohen’s Kappa was 0.965 (96.9% agreement, *P*<0.001). For the barriers question data, Cohen’s Kappa was 0.904 (91.7% agreement, *P*<0.001).

#### What would make a health check easier to access?

**Figure 1.**
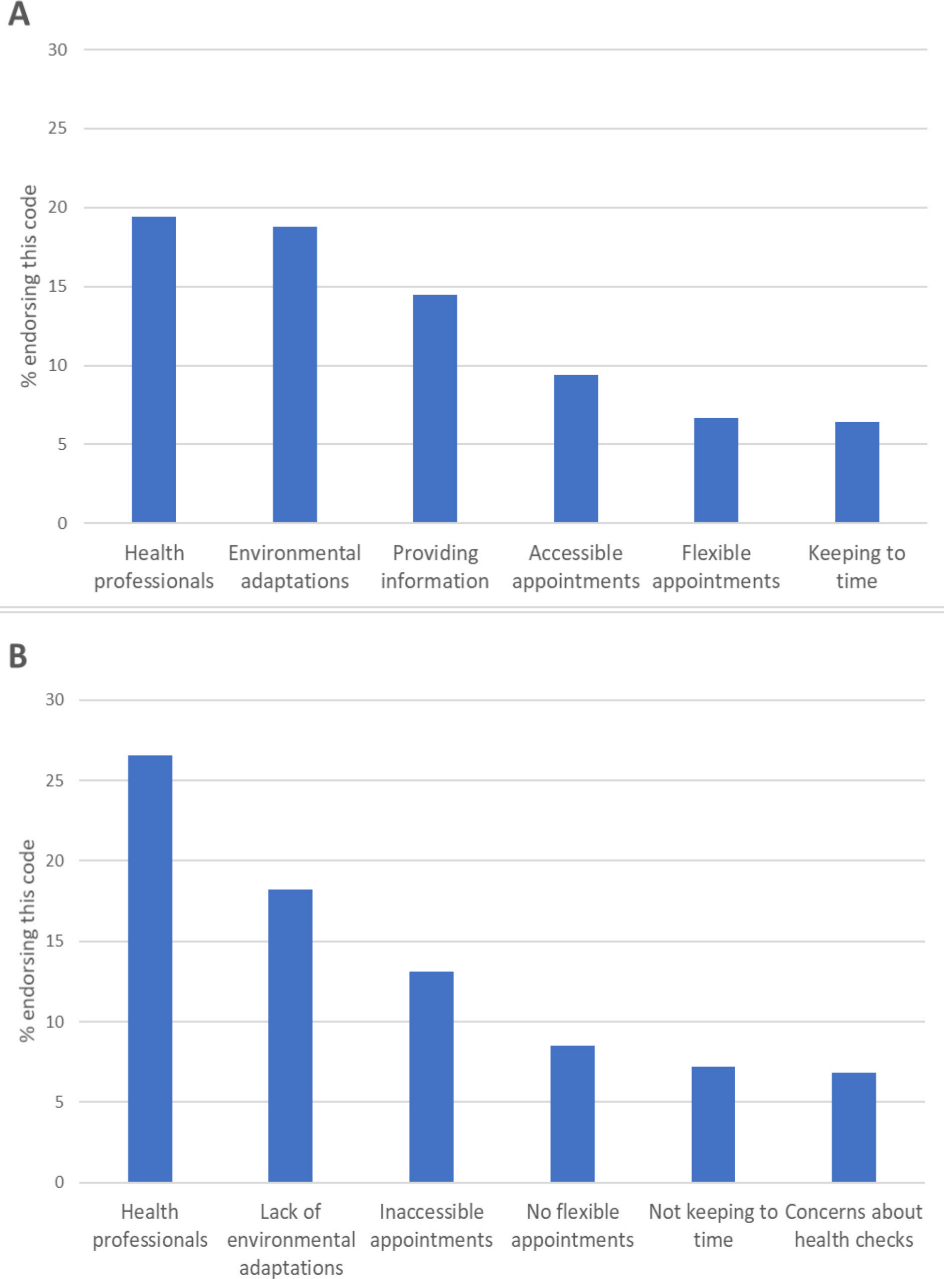
Representation of the categories for (**A**) 'What would make a health check easier to access?' and (**B**) 'What would make a health check more challenging to access?'

Supplementary Table S1 lists the response categories and illustrative anonymised quotations for each of the categories. The most frequent responses about what would make a health check easier were: factors relating to health professionals, such as a familiar clinician, and/or someone who knows about autism (19.5%, *n* = 171); environmental factors such as low-level sensory stimulation, especially in the waiting room (18.9%, *n* = 166); and providing information about the health check, such as who the participant will be seen by, photographs of the venue and staff, and information about what will happen (14.6%, *n* = 128) (see Figure 1). Responders also identified specific requirements of the health check (5.0%, *n* = 44), including offering adjustments, doing health checks online, health professionals reviewing notes beforehand, and having a written summary detailing the outcomes of the appointment.

#### What would make a health check more challenging to access?

The responses were consistent with those reported above, with health professionals (26.5%, *n* = 215) and environmental factors (18.1%, *n* = 147) listed as the biggest potential challenges to a health check (see Figure 1). Responses indicated that health professionals could make health checks more challenging for reasons by being 'patronising' or 'not knowledgeable about autism'. Other factors included inaccessible appointments (for example, inflexibility with appointment times and locations; 13.1%, *n* = 106) and factors related to the organisation of the appointment, such as a lack of support or unclear communication. Only 6.8% (*n* = 55) reported concerns about the health check appointment itself (for example, being put on the spot to answer questions, being pressured to attend, long forms to complete on the day and undertaking physical assessments without seeking consent, such as measuring blood pressure).

## Discussion

### Summary

The study represents a first step in the authors' research programme to co-design and evaluate a health check for autistic adults, and to address this aim of the UK *NHS Long Term Plan*.^23^ The findings suggest strong support from autistic people for a health check and provide useful information for its design and delivery in primary care. The majority thought a health check should be available for all autistic people, irrespective of intellectual ability, and include consideration of personalised reasonable adjustments to improve access to health care, with flexible delivery methods to maximise acceptability.

Open-text responses identified some key factors relevant to a health check for autistic people, and these could apply more generally to healthcare encounters. Specifically, flexible ways of booking appointments (for example, booking online avoiding a phone call) and providing advance information about the health check were perceived as facilitators for access. Incorporating these factors into the development of a health check for autistic adults may increase uptake.

### Strengths and limitations

The large survey included autistic people from a wide age range including 61 people aged ≥61 years (13.3%), a group whose views are often missing from the autism literature.^29,30^ Through ASC-UK recruitment, it was possible to include a large number of female participants in this study. The age and sex distribution allowed the authors to investigate sex and age effects, although both factors could not be considered simultaneously. Demographic information, including sex, was gathered from ASC-UK and the response options were based on advice received from autistic people. As such, the authors are not able to comment on whether the findings are generalisable to people who do not report as male or female; this group may face additional challenges accessing health care and more research is needed to investigate what maximises good healthcare access. Finally, proxy responders were included in the analyses, but the views of those with intellectual disability were under-represented; further exploration is needed to help inform health check implementation. The open-text responses were necessarily brief; interviews and focus groups focusing on this topic are also part of the health check development process and are reported elsewhere.^31^

### Comparison with existing literature

The findings are in agreement with previous literature that finds some factors impact negatively on healthcare access.^11,12,15^ Autistic adults emphasised the importance of health professionals having a good understanding of autism, as is also identified elsewhere.^12^ A lack of professionals’ understanding may lead to autistic people being less satisfied with patient–provider communication,^32^ which could be to the extent that health conditions are not identified.^30^ A final consideration is continuity of GP or practice nurse seen. This is important in health care generally,^33^ but may be particularly relevant for autistic people.^34^

### Implications for research and practice

Considering the aims of the NHS Long Term Plan and autistic adults’ views here, evaluating a health check specifically designed for autistic adults is an appropriate next step. A subsequent trial of its clinical and cost effectiveness is planned, and if demonstrated, the study results will be important for those considering implementation in primary care. Future studies to investigate health check use for autistic people in other countries will be important, as provision will need to be made appropriate for other health systems.
